# A Family With a Complex Phenotype Caused by Two Different Rare Metabolic Disorders: GLUT1 and Very-Long-Chain Fatty Acid Dehydrogenase (VLCAD) Deficiencies

**DOI:** 10.3389/fneur.2020.00514

**Published:** 2020-06-23

**Authors:** Olimpia Musumeci, Edoardo Ferlazzo, Carmelo Rodolico, Antonio Gambardella, Monica Gagliardi, Umberto Aguglia, Antonio Toscano

**Affiliations:** ^1^Unit of Neurology and Neuromuscular Disorders, Department of Clinical and Experimental Medicine, University of Messina, Messina, Italy; ^2^Institute of Molecular Bioimaging and Physiology, National Research Council, Catanzaro, Italy; ^3^Department of Medical and Surgical Sciences, Magna Græcia University, Catanzaro, Italy; ^4^Regional Epilepsy Centre, “Bianchi-Melacrino-Morelli” Great Metropolitan Hospital, Reggio Calabria, Italy

**Keywords:** GLUT1-DS, VLCAD, rhabdomyolysis, metabolic myopathy, genetic “double-trouble”

## Abstract

GLUT1 Deficiency Syndrome (GLUT1-DS) is a rare and potentially treatable neurometabolic condition, caused by a reduced glucose transport into the brain and clinically characterized by an epileptic encephalopathy with movement disorders. A wide inter-intrafamilial phenotypic variability has been reported. Very-long-chain acyl-CoA dehydrogenase (VLCAD) deficiency is an inherited metabolic disorder of mitochondrial long-chain fatty acid oxidation (FAO) with also a variable age of onset and clinical presentation including cardiomyopathy, hypoketotic hypoglycemia, and liver disease. Sometimes, VLCAD manifests later with a prevalent muscle involvement characterized by exercise intolerance and recurrent rhabdomyolysis. We report a 40-year-old man with mild mental retardation and sporadic choreo-athetoid movements, who complained of recurrent episodes of rhabdomyolysis triggered by exercise or fasting since his twenties. His 15-year-old son had a psychomotor developmental delay with episodes of drowsiness mainly at fasting and exercise-induced choreo-athetoid movements but no history of pigmenturia. Clinical and laboratory findings in the son suggested a diagnosis of GLUT1-DS confirmed by *SCL2A1* genetic analysis that revealed a heterozygous mutation c.997C>T (p.R333W) that was also found in the proband. However, the presence in the latter of recurrent exercise-induced rhabdomyolysis, never reported in GLUT1-DS, implied a second metabolic disorder. Increased plasma C14:1-carnitine levels and the identification of two known heterozygous mutations c. 553G>A (p.G185S) and c.1153C>T (p.R385W) in *ACADVL* confirmed the additional diagnosis of VLCAD deficiency in the proband. Nowadays, there is an increasing evidence of “double trouble” cases of genetic origin. Consequently, when atypical features accompany a known phenotype, associated comorbidities should be considered.

## Introduction

Glucose transporter type 1 deficiency syndrome (GLUT1-DS) is a rare and potentially treatable condition, caused by defect of GLUT1, encoded by SLC2A1 (OMIM ^*^138140) on chromosome 1, that transports glucose into the brain through the blood–brain barrier. GLUT1–DS deficiency (OMIM #606777) is caused by mutations in SLC2A1, which result in hypoglycorrhachia leading to cerebral energy deficiency (1). GLUT1 is highly expressed in endothelial cells of erythrocytes and blood–brain barrier but less in adipose and muscular tissues (2). GLUT1-DS manifests with an epileptic encephalopathy and was firstly reported by De Vivo in children with intractable epilepsy, ataxia, spasticity, and mental retardation (3). Cognitive impairment may vary from mild to severe (4). Thereafter, phenotypic spectrum greatly widened including paroxysmal movement disorders and variably severe seizure disorders. Non-classical phenotype also includes adult cases and variants without epilepsy but neuromuscular symptoms have never been reported. (5). Seizures and paroxysmal movement disorders can be triggered by fasting and physical exercise and improve after meals. In the majority of cases, the disease is associated with heterozygous “*de novo*” mutations but may also be transmitted as an autosomal dominant or recessive trait (6).

Rhabdomyolysis can be caused by several acquired or inherited etiologies. Most of the inherited causes of rhabdomyolysis are inborn errors of metabolism that result in impaired energy production due to defects of glycogen or lipid metabolisms. Patients complain of recurrent episodes accompanied by exercise intolerance, muscle aches, and contractures; a positive family history may suggest an underlying genetic disorder (7, 8).

Very-long-chain acyl-CoA dehydrogenase (VLCAD) deficiency (OMIM 609575) is an autosomal recessive disorder of mitochondrial fatty acid oxidation (FAO) (9). The enzyme is located in the mitochondrial inner membrane and catalyzes the dehydrogenation of long-chain acyl-CoA esters of 12–18 carbons, which is the first step of FAO. VLCAD is encoded by *ACADVL*, which comprises 20 exons (10). VLCAD deficiency can present with a variety of clinical symptoms and different severity. Classically, three phenotypes have been recognized: (a) early infantile onset characterized by hypertrophic or dilated cardiomyopathy, pericardial effusion, hypotonia, hepatomegaly, or severe hypoketotic hypoglycemia, often fatal because cardiomyopathy and arrhythmias such as ventricular tachycardia, ventricular fibrillation, and/or atrioventricular block; (b) childhood-onset hypoglycemia type and hepatomegaly induced by preceding infections or long fasting during early childhood characterized by a delayed onset, lower mortality, and rare or absent cardiomyopathy; (c) juvenile/adult-onset later-onset type mainly presenting with episodic symptoms, consisting of skeletal muscle symptoms such as myalgia, muscle contractures, weakness, exercise intolerance, and/or rhabdomyolysis during physical exercise or illness (11). The implementation of newborn screening programs for FAODs allowed for an early diagnosis with detection of several cases at a presymptomatic stage of the disease but also increased the recognition of either milder phenotypes or asymptomatic individuals. The disease is due to *ACADVL* mutations that were firstly identified in 1995: since then, several variants have been reported with unclear genotype–phenotype correlation (12).

We herein report a family with VLCAD/GLUT1 deficiencies. The proband presented with exercise-induced rhabdomyolysis as main clinical manifestation whereas, his son had a prominent central nervous system involvement. Biochemical and genetic investigations revealed a combination of two rare metabolic disorders, emphasizing the importance of a diagnostic challenge in similar cases.

## Case Presentations

**Figure 1:**
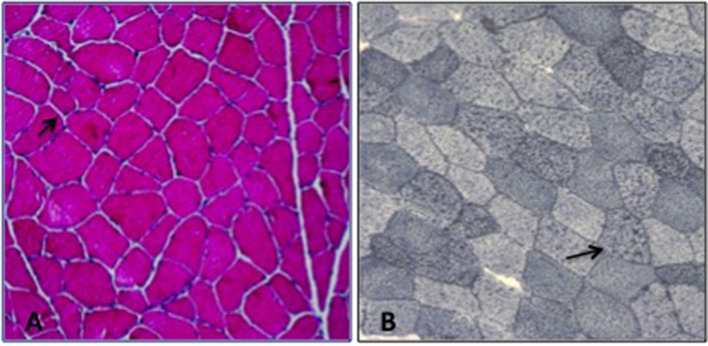
Muscle biopsy; **(A)** H&E: increased fiber size variability, **(B)** Sudan Black: lipid storage in several myofibers (arrow).

Patient 1. The proband is a 40-year-old man, who complained, since 25 years of age, of recurrent episodes of myalgia and generalized muscle weakness associated with dark urines, usually occurring after intense physical exercise or prolonged fasting. At clinical examination, the patient revealed low-set ears, microcephaly, dysarthria, gait unsteadiness, and sporadic choreo-athetoid movements. Neuropsychological profile disclosed a mild cognitive impairment (Wechsler Adult Intelligence Scale: total IQ at 54). Brain magnetic resonance imaging (MRI) and electroencephalography (EEG) were normal. Electrocardiogram and echocardiography were normal. There was no evidence of hepatomegaly or hypoglycemia. Blood examination during a metabolic crisis induced by exercise, revealed marked serum CK increase up to 75,000 U/L [reference range (r.r.), 0–200 U/L], lactate dehydrogenase (LDH) at 590 U/L (r.r., 47–140), alanine aminotransferase (ALT) at 492 U/L (r.r., 10–40), and aspartate aminotransferase (AST) at 1728 U/L (r.r., 7–56); 24 h myoglobin urinary excretion was 285.000 μg/L [reference values (r.v.), <50 μg/L]. Vitamins D, E, B12, folic acid serum levels and thyroid function parameters were normal. During intercritical periods, CK, AST, ALT, and LDH were normal. Measurement of plasma acylcarnitines by tandem mass spectrometry revealed accumulation of tetradecenoylcarnitine C14:1 (1.5; r.r., 0.02–0.19 μmol/L); C14:1/C12:1 ratio was 8.3 (r.v., <4), suggesting a VLCAD deficiency (13). Electromyography (EMG) showed a myopathic pattern. A tibialis anterior muscle biopsy was performed at 40 years of age and showed mild unspecific changes with a mild lipid storage (Figures 1A,B). Biochemical analysis on muscle homogenate, including carnitine-palmitoyl-transferase II (CPT2) and glycolytic enzymes activities, did not show any defect. Mitochondrial respiratory chain enzyme activities were normal but muscle Coenzyme Q10 (CoQ10) level was mildly reduced (16 μg/g muscle; r.v., 25 ± 3).

Thereafter, the patient stopped performing prolonged physical exercise, so avoiding further episodes of rhabdomyolysis.

Patient 2. The 15-year-old son manifested with drug-resistant epilepsy since the age of 2 years. Seizures occurred at daily frequency, and they lasted from minutes to hours and were characterized by drowsiness, psychomotor slowing, and head drop. These episodes mainly appeared before meal, disappeared after sugar or food intake, and were not responsive to treatment with lamotrigine and topiramate. Around the age of 6 years, he started to present with lower limb choreo-athetoid movements that were provoked by prolonged exercise and disappeared after 30–60 min of rest. Clinical examination revealed microcephaly and low-set ears as well as moderate spastic tetraparesis. Neuropsychological examination evidenced moderate cognitive impairment (Wechsler Intelligence Scale for Children: total IQ at 40). Brain MRI only showed mild hyperintensities of periventricular white matter predominant over posterior head regions. A wide hematological metabolic screening was normal. EEG, recorded during fasting, showed almost continuous, diffuse, spike and polyspike and wave discharges that disappear after food or glucose intake. CSF analysis revealed hypoglycorrhachia (40 mg/dl) with CSF/serum glucose ratio 0.4 (r.v., >0.55). Molecular analysis of *SCL2A1* showed a pathogenic heterozygous mutation c.997C>T (p.R333W), already reported in the literature as frequent mutation associated with GLUT1-DS (14). However, the patient refused the ketogenic diet. Molecular analysis was then extended to the proband confirming the same genotype.

**Figure 2:**
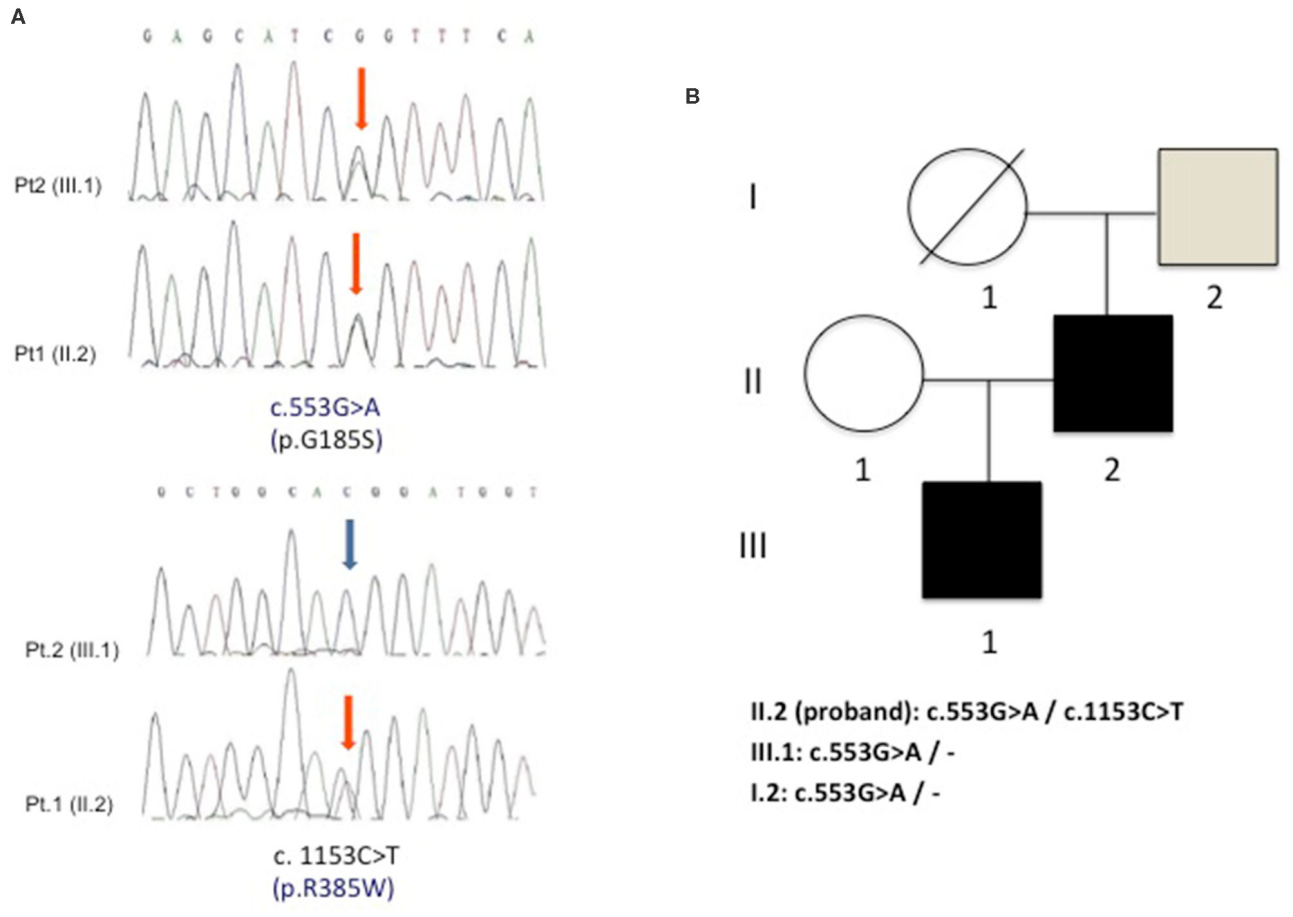
**(A)** Electropherogram of *ACADVL* showing the two heterozygous substitutions c.553G>A (p.G185S) and c.1153C>T (p.R385W) in the proband (pt. 1) and the presence of only c.553G>A (p.G185S) in the son (pt. 2). **(B)** Family tree shows the segregation of the two G185S and R385W mutations in the analyzed family members.

Since recurrent rhabdomyolysis has never been reported among GLUT-1-DS clinical manifestations, a second disease was suggested in the proband. Molecular study of *ACADVL* identified in the proband two known pathogenic heterozygous mutations c. 553G>A (p.G185S) and c.1153C>T (p.R385W), whereas his son carried only the heterozygous p.G185S variant. The asymptomatic father of the proband only showed a p.G185S mutation in heterozygous state, while it was not possible to test his mother because she died many years earlier (Figure 2). No consanguinity was reported in the family.

All clinical and laboratory findings are summarized in Table 1.

**Table 1:** Clinical, biochemical, and genetic findings.

**Case**	**Sex/Age**	**Age at onset**	**Clinical symptoms**	**Triggers**	**Laboratory tests**	**EEG**	**EMG**	**Brain MRI**	**Muscle biopsy**	**SCL2A1 mutation analysis**	**ACADVL mutation analysis**
1	M/40	25 years	Episodes of myalgia, muscle weakness, and myoglobinuria Sporadic choreo-athetotic movements	Physical exercise Fasting	↑CK 75000 UI/L ↑ALT 492UI/L ↑ AST 1728 UI/L ↑ Urinary myoglobin 285,000 μg/L ↑ C14:1 1.5 μmol/L ↑ C14:1/C12:1 ratio: 8.3 CSF/serum glucose ratio: n.p.	Normal	Myopathic pattern	Normal	Increased fiber size variability Slight lipid storage in some fibers	c.997C>T/– p.R333W/–	c.553G>A/c.1153C>T p.G185S/p.R385W
2	M/15	2 years	Intellectual disability Generalized seizures Choreo-athetoid movements	Physical exercise Fasting	CK, ALT, AST: normal Urinary myoglobin: n.p. Acylcarnitines profile: normal↓ CSF/serum glucose ratio: 0.4	Generalized irregular 2–4 Hz spike-wave discharge	Normal	Mild posterior periventricular white matter alterations	n.p.	c.997C>T/– p.R333W/–	c.553G>A/– p.G185S/–

*CK, creatine kinase; ALT, alanine aminotransferase; AST, aspartate aminotransferase; CSF, cerebrospinal fluid; EEG, electroencephalogram; EMG, electromyography; MRI, magnetic resonance imaging; n.p: not performed. ↑, increased value; ↓, decreased value*.

## Discussion

Inherited metabolic disorders are rare conditions characterized by clinical and genetic variability; frequently atypical features are reported as an expansion of the known phenotype.

In this paper, we report on a family where some atypical aspects were then explained by a defined combination of two rare metabolic defects, GLUT1 and VLCAD deficiencies, that determined an impairment of energy availability in the brain or in skeletal muscle. Fasting, physical exercise, and other catabolic conditions increased energy demand and are known triggers of both conditions.

Since the first description of GLUT-1 DS, a wide phenotypic spectrum has been reported. The classical encephalopathy in GLUT1 deficiency is characterized by infantile-onset pharmacoresistant epilepsy, intellectual disability, microcephaly, and complex movement disorders. Non-classical phenotypes include exercise-induced paroxysmal dyskinesia, “atypical” childhood absence epilepsy, and myoclonic astatic epilepsy; seizures may never occur (14–17). The present family showed an intrafamilial phenotypic variability, already described in GLUT1-DS (18); the son manifested a classical phenotype with a developmental and epileptic encephalopathy whose diagnosis was supported by biochemical and genetic analysis, whereas his father showed only few typical GLUT1-DS features such as mild cognitive impairment, microcephaly, gait unsteadiness, and mild movement disorders (choreo-athetoid features) but no seizures, even though he harbored the same R333W mutation in *SCL2A1* confirming the GLUT1-DS diagnosis.

On the other hand, in our proband, recurrent rhabdomyolysis, induced by physical efforts or fasting, was the prominent clinical aspect suggesting a metabolic muscular disorder. Inherited metabolic myopathies include several enzymatic defects in different pathways such as glycogen catabolism (glycogenolysis and glycolysis), FAO, or mitochondrial respiratory chain and oxidative phosphorylation, and can present with episodes of exercise intolerance, contractures, myalgia, and myoglobinuria (19). A long series of biochemical and genetic investigations allowed reaching the diagnosis of VLCAD deficiency as a second disease explaining this “challenging” phenotype.

Within FAODs, VLCAD is the most frequently reported condition, but rarely other genetic defects have been described (7).

The expansion of newborn screening programs for FAODs increased substantially the number of VLCAD cases, even though the diagnosis needs to be confirmed by *ACADVL* genetic analysis and/or functional studies on fibroblasts or lymphocytes. So far, more than 300 mutations in *ACADVL* have been reported (http://www.hgmd.cf.ac.uk/ac/gene.php?gene=ACADVL).

The herein described patient carried two mutations (p.G185S and p.R385W), both already reported (20, 21) in other individuals. The *ACADVL* c.553G>A; p.G185S variant also known as G145S, has been described in association with another pathogenic variant (21, 22). Functional characterization of patient fibroblasts carrying this variant indicates a decrease in VLCAD protein levels resulting in significantly lower enzymatic activity compared to wild type (20, 23). The glycine at position 185 is highly conserved and is located in the substrate binding cavity (24). Prediction algorithms (PolyPhen-2, SIFT) predict that the p.G185S variant has an impact on VLCAD protein structure.

The *ACADVL* c.1153C>T; p.R385W variant, also reported as R345W, has been described in several VLCAD individuals (21, 25); the arginine at codon 385 is moderately conserved, and prediction programs (PolyPhen-2, SIFT) indicate that this variant is deleterious. Recombinant VLCAD protein harboring the p.R385W substitution showed a reduction in enzyme activity compared to wild type (26).

Moreover, it is interesting that our proband showed a mild reduction of muscle CoQ10 content, which could be likely considered as a secondary phenomenon. An association between GLUT1-DS and a mild CoQ10 deficiency has been reported in a 15-year-old girl with truncal ataxia, nystagmus, dysarthria, and myoclonic epilepsy (27) but other studies, investigating the association of GLUT1-DS and CoQ10 deficiency in a larger cohort of GLUT1-DS patients, do not show other similar cases (28). On the other hand, CoQ10 deficiency has been found in patients with lipid storage myopathy due to ETFDH deficiency (29) and in a patient with VLCAD deficiency (30). CoQ10 acts as a direct acceptor of electrons from ETF and it is likely that, in FAOD, a dysfunction of reducing enzymes could down-regulate its biosynthesis (31).

An early recognition of GLUT1-DS but also of VLCAD deficiency is crucial to correctly manage these patients. Avoidance of trigger events such as prolonged fasting and intense physical efforts is recommended in both conditions (12, 16). GLUT1-DS patients may greatly benefit from a ketogenic diet, with efficacy on seizures and movement disorders (16, 32). However, the son of our proband refused ketogenic diet because of strong concerns over diet restriction. Treatment options for VLCAD deficiency include diet low in long-chain fatty acids supplemented with medium-chain triglycerides or L-Carnitine at low doses but their use is still controversial and novel therapeutic agents as triheptanoin or bezafibrate are currently under investigation (11).

Avoiding physical exercise was useful to prevent episodes of myoglobinuria in our proband but he refused diet interventions.

## Conclusions

In conclusion, the combination of these two rare metabolic disorders gave rise to a quite peculiar clinical phenotype in this unique family. The increasing evidence of “double trouble” cases suggests that, when a known phenotype is “complicated” by atypical features, it is worthwhile to search for a more complete diagnosis.

## Ethics Statement

Written informed consent was obtained from the patient for himself and for his son, for the publication of any potentially identifiable images or data included in this article.

## Author Contributions

OM, EF, UA, and AT participated in the design of the study. OM, EF, and AG collected all clinical data and performed neurological examinations. OM and MG provided all of the biochemical and molecular data. CR did the histopathological investigations. All authors helped in drafting the manuscript and read and approved the final manuscript.

## Conflict of Interest

The authors declare that the research was conducted in the absence of any commercial or financial relationships that could be construed as a potential conflict of interest.
